# Bone Mineral Density and Bone Turnover Markers in Postmenopausal Women Subjected to an Aqua Fitness Training Program

**DOI:** 10.3390/ijerph16142505

**Published:** 2019-07-13

**Authors:** Krystian Wochna, Alicja Nowak, Anna Huta-Osiecka, Katarzyna Sobczak, Zbigniew Kasprzak, Piotr Leszczyński

**Affiliations:** 1 Department of Swimming and Water Lifesaving, Poznan University of Physical Education, Królowej Jadwigi 27/39, 61-871 Poznań, Poland; 2 Department of Hygiene, Poznan University of Physical Education, Królowej Jadwigi 27/39, 61-871 Poznań, Poland; 3 Department of Rheumatology and Rehabilitation, Poznan University of Medical Sciences, 28 Czerwca 1956 r., 61-545 Poznań, Poland

**Keywords:** aqua fitness, bone mass, bone metabolism, postmenopausal women

## Abstract

The purpose of this study was to analyze the influence of aqua fitness training in deep water on bone tissue. The study was performed with 18 postmenopausal women separated into two groups: training and control groups. Before and after the training program, the hip and spine areal bone mineral density were measured along with the biochemical parameters of serum concentration of osteocalcin (OC) and C-terminal telopeptide of type I collagen (CTX). The most significant effect was found in differences between the two groups of women in terms of femur strength index (*p* < 0.05) during the period of the training program. The study demonstrated that an aqua fitness training program caused favorable changes in femur strength index in postmenopausal women, and this kind of exercise could be a useful form of physical activity for postmenopausal women.

## 1. Introduction

Osteoporosis is currently a serious, chronic public health problem worldwide [1,2]. Intervention with exercise is important to prevent morbidity [3] and to improve the quality of life for women with postmenopausal osteoporosis [4,5].

Although conflicting results exist regarding the effects of exercise on bone mass in postmenopausal women, systematic reviews indicate that exercise may result in clinically relevant benefits to BMD [6,7]. The effects of physical activity on bone tissue are primarily linked to the mechanisms of mechanical loading. Several findings indicate that gravitational loading is essential for bone homeostasis [8]. With respect to the source of loading, some authors divide exercises into those with high impact and those with low impact. Exercises in the water environment stimulate the skeleton almost exclusively through muscle loading because they involve little or no impact with the ground [9]. Studies conducted on young swimmers have demonstrated negligible effects on the skeleton and bones as a response to swimming [10]. Moreover, there is no confirmed relationship between bone mineral density and individual swimming techniques. Extended periods of immersion in water leads to a decreased load on weight-bearing bones [11].

A water training program incurs a lower risk of traumatic fractures and impact on the joints when compared to land-based exercise. In recent years aqua fitness training has become popular, especially among the adult population, because water-based exercises can be an effective way to improve physical function [12,13]. Meta-analysis focused on effectiveness of water-based exercise (WBE) on bone health of middle-aged and older adults revealed significant differences between WBE and sedentary control group in favor of WBE for changes in bone mineral density (BMD) at the lumbar spine and femoral neck. However, the authors found no significant difference between WBE and land based exercise groups for changes in femoral neck BMD [13].

Although there are several reports on the effects of exercise in water on bone metabolism in postmenopausal women, [14,15,16,17] we found no studies in which the protocol was implemented in deep water. With regard to the depth of immersion, the greater it is, more force is required to move the body forward against water resistance [18]. Moreover, despite BMD measurement being the most frequently used method to diagnose the reaction of bone on mechanical loading, bone microarchitecture and geometric properties provide more information about bone response [19]. Therefore, the purpose of the study was to analyze the influence of aqua fitness training in deep water on bone mineral density, bone strength index and biochemical indices, using materials that increase water resistance on the skeleton.

## 2. Material and Methods

### 2.1. Participants

The study was performed with 18 postmenopausal women (aged between 54 and 65 years), all of whom declared they were in good health. Participants with recent infections, inflammatory disorders, diabetes mellitus, osteoporosis and fractures in the past 12 months, or who were undergoing hormone replacement therapy were not included in the study. Subjects were separated into two groups: the training group (T) consisting of nine women who participated in the aqua fitness program and the control group (C) consisting of nine women who did not participate in any systematic physical activity during the research period. The participants were matched, as closely as possible, with respect to age, duration of menopause, and body mass index (BMI). All subjects declared that they did not have a history of professional sports. All participants gave written informed consent to participate in the study program. The study protocol was approved by the Ethics Committee at the Poznan University of Medical Sciences (code no. 1224/17).

### 2.2. Training Program

Participants from the training group performed two 45-minute aqua fitness classes per week over a 6-month period. All classes were prepared and conducted by the same instructor, a member of the research team. The training included exercises in deep water (to the neck line) using equipment that increased the body contact area with the water such as pool noodles, aqua dumbbells, water gloves, aqua Betomics, balls, resistance bands, leg weights and aqua discs. Exercises were performed using simple choreography set to music. All participants carried water belts that enabled them to keep the body in a vertical position. The resistance was controlled by positioning the hands and feet. The upper limbs moved in every plane, mostly in the frontal position, with either open fingers or fists. This facilitated the maintenance of the correct position and utilized the biophysical properties of water fully. Upper limb movements were coordinated with other basic components of aqua fitness training, such as: balance, sculling, walking and kicking.

### 2.3. Bone Density Measurements

Areal bone mineral density (aBMD) was measured using dual X-ray absorptiometry (DXA) on the whole body, the left hip (total hip and neck) and the lumbar spine (L_1_–L_4_). The DXA measurements were acquired using a Lunar Prodigy Advance densitometer (General Electric, USA). The DXA measurements were expressed as areal bone mineral density (aBMD, g/cm^2^) and T-score. Total fat, lean body mass, android and gynoid fat tissue were determined. All scans were taken by the same technician using the same device, which was calibrated daily. Quality control of the DXA scanner was performed in accordance with the manufacturer’s instructions, and scan analyses were performed using integrated software according to the manufacturer’s recommendations. The strength index of the left hip was automatically calculated using the ratio of estimated compressive yield strength of the femoral neck to the expected compressive stress of a fall on the greater trochanter [19]. This algorithm considers the shape of the proximal femur as well as the cross-sectional moment of inertia in the estimate [20].

### 2.4. Biochemical Analysis

Fasting morning blood was collected from the antecubital vein between 07:30 a.m. and 10:00 a.m. and was centrifuged at 4000 rpm at 4 °C. The serum was prepared from the sample and stored at –70 °C. Serum concentrations of the bone turnover markers osteocalcin (OC, Human Osteocalcin Instant ELISA, produced by Bender MedSystems, Vienna, Austria, assay sensitivity 0.2 ng/mL) as a bone formation marker and collagen type I cross-linked C-telopeptide (CTX-I, Serum CrossLaps ELISA, produced by Immunodiagnostic Systems, Boldon, UK, assay sensitivity 0.02 ng/m:) as a bone resorption marker were determined using an enzyme-linked immunosorbent assay (ELISA) method.

### 2.5. Statistical Analysis

The data are presented as mean, standard deviation (SD), median and interquartile range. The normality of distributions was verified using the Shapiro–Wilk test. The *t*-test and Mann–Whitney U test were employed for normally and non-normally distributed variables, respectively, to evaluate the significance of differences between the groups. A 2 × 2 (group × time) repeated-measures ANOVA was used to evaluate the influence of the training program on the assessed indices. The Pearson analysis for normally distributed variables and Spearman’s rank analysis for non-normally distributed variables were used to calculate correlation coefficients. Statistical significance was set at an alpha of 0.05 for all statistical procedures. The obtained results were analyzed statistically using the Dell Inc. (2016) Dell Statistica (data analysis software system), version 13. software.dell.com (Dell Inc., Round Rock, TX, USA).

## 3. Results

There was no significant difference between the training and control groups with respect to age (mean ± SD: 58 ± 3.27 vs. 60 ± 3.37 years, respectively) or duration of menopause (9.6 ± 4.46 vs. 11.6 ± 6.14 years, respectively), as the result of matching both groups. There were no significant differences between the investigated groups with respect to the variables measured before the study (descriptive statistics of these variables are presented in Table 1 and Table 2, *p* ≥ 0.05). While analyzing the group’s impact on the variables the significant difference was found for CTX concentrations (*p* = 0.0105, power = 0.78), which in women from the training group were lower than in women from the control group (mean ± SD: 0.37 ± 0.11 ng/mL vs. 0.54 ± 0.30 ng/mL, respectively). No group effects were found for the other measured indices. While analyzing the time’s impact on the variables (between both terms of the study) no effects were found.

**Figure 1:**
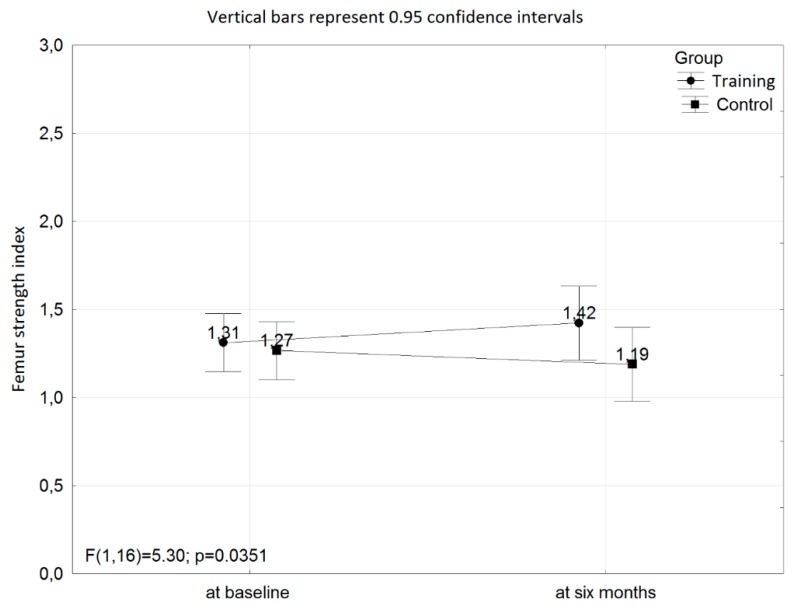
The interaction with respect to the femur strength index at baseline and after six months of the training program between the training and control groups.

Table 1 and Table 2, and Figure 1 present the results of somatic features, areal bone mineral density, femur strength index and serum concentrations of biochemical indices before and after the intervention in both investigated groups of women.

The interaction with respect to the femur strength index at baseline and at six months of the training program between the training and control groups was found (*p* = 0.0351; power = 0.58; Figure 1).

Within the training group, the correlation analysis did not show any relationship between changes (Δ) in femur strength index value after the intervention with somatic features, serum concentrations of bone turnover markers or bone mass indices measured before the training program. Moreover, no relationships were found between changes (Δ) in femur strength index value or CTX concentrations with changes of other indices of bone mass or body composition.

For the control group, negative correlations were found between changes (Δ) in OC concentration and BMI values and changes in OC concentration and T-score for total femur values during the study period (r = −0.71, *p* = 0.046 and r = −0.84, *p* = 0.009, respectively).

## 4. Discussion

In the present study, we noticed a significant interaction between the investigated groups of postmenopausal women and the time of the intervention with respect to femur strength index, which is relevant in light of the fact that hip fractures are a mortality risk [21]. However, we found no significant influence of the aqua fitness program on bone mineral density values measured on the whole body, the left hip or the lumbar spine regions. Despite the many studies showing that physical activity is an important factor positively stimulating bone metabolism during all life stages, water-based exercises may induce varied responses in the skeleton. Most studies conducted in young swimmers showed similar BMD values to sedentary controls [22]. However, Gómez-Bruton et al. [23] in a review focusing on physical activity in water and bone health noticed that in a population of adults over 40 years of age, swimming reduced the rate of bone mass loss accompanying ageing. They suggested that these differences could be sport-specific or could have occurred due to the more active lifestyle reported by swimmers.

In our experiment, we found no significant influence of the training program on BMD values, but the exercises were performed in deep water and lasted only six months. However, we observed a significant impact on the femur strength index, which may be explained by the use of special equipment that increases the surface of contact with water and the resistance exerted on the skeleton. In postmenopausal women, there are several studies that have investigated the influence of training in a water environment on skeletal health in both healthy [17] and osteoporotic subjects [24]. Balsamo et al. [17], conducted a study in a population of women with a mean age of approximately 55 years. The authors demonstrated that women who took part in aquatic weight-bearing exercises (*n* = 22) for at least one year had a higher BMD in the whole body, whole hip and lumbar spine L_2_–L_4_ compared to controls. However, the women in that study attended the classes at least three days per week, and their exercises were focused on major muscle groups, with movements of pushing, pulling and jumping. The authors suggested that in-water weight-bearing exercise could be a valuable non-pharmacological alternative to prevent BMD loss in postmenopausal women. Murtezani et al. [24], tested a population of women with osteoporosis with a mean age of 59.7 years (*n* = 30). Women who performed aquatic exercises were compared to women who participated in an exercise program on land. The training program lasted ten months and was conducted three times a week. Water exercises took place in shallow water with no equipment. The authors demonstrated that both land and aquatic exercises had beneficial effects on lumbar spine BMD in osteoporotic women, but that land classes seemed to be more effective.

Despite BMD measurement being the most frequently used method to diagnose osteoporosis, other factors (such as bone microarchitecture and geometric properties) provide information about fracture risk. Faulkner et al. [19], in a study conducted on a large population of women over 50 years of age, compared femoral bone density, structure and strength assessments obtained from DXA measurements and concluded that these indices are significant independent predictors of hip fractures. X-ray based technology will become available to estimate components of bone strength and that it may play a role in fracture risk assessment [25]. Faulkner et al. [19], proposed that hip axis length is one of the best geometric measures indicating hip fracture risk for females, independently of BMD at the femoral neck. This was indicated by previous findings that geometric measures and strength indices are important in femoral neck strength prediction. Yang et al. [26,27] in a prospective case-cohort study found that the estimation of femoral strength from finite element analysis of DXA scans was an independent predictor and performed at least as well as femoral neck BMD in predicting the incidence of hip fractures in postmenopausal women. Wendlova [28] compared measurements of femur strength index with BMD variable values in the total hip area. She reported that variable values of femur strength index may better indicate the probability of fractures in the femoral neck area.

The biochemical indices applied in our study [i.e., markers of bone synthesis (OC) and its degradation (CTX)] make it possible to estimate the current status of bone metabolism. Bone turnover markers (BTMs) are biomarkers released during bone remodeling by osteoblasts or osteoclasts. BTMs are increased after the menopause, and one potential clinical application of these indices of skeletal metabolism is in assessing fracture risk [29]. We found no significant effect of the training program with respect to BTMs. The increase in CTX levels in both groups of women during the time of the intervention may indicate the loss of bone mass in the postmenopausal period. This phenomenon is also confirmed by the negative correlation between changes in OC concentration and T-scores of total femur values during the study period in the control group. On the other hand, OC participates in metabolic processes, such as glucose homeostasis and exercise capacity, which are modified during systematic physical activity. In a feed-forward loop mechanism, metabolic improvement can affect the release of OC from the bone [30]. Moreover, the effect of the decrease in skin vitamin D synthesis connected with the fall in ultraviolet B radiation could also play a role (the study was conducted from September to March) [31].

Our study has demonstrated the beneficial effects of a water training program on bone strength, however, the limitation is the small sample size. Physical activities in deep water may be a good alternative to exercise on the ground due to the low impact on joints and greater psychological comfort for older individuals, especially for overweight people who often suffer from joint pain. Furthermore, other studies have underlined additional positive effects of deep water training regarding metabolic and physiological variables (running performance, VO_2_max, heart rate, lactate threshold) as well as neuromuscular functions [18,32].

## 5. Conclusions

The study showed that an aqua fitness training program for postmenopausal women led to an increase in bone strength index. However, we observed the lack of changes in levels of somatic indices and bone turnover markers, therefore, in the future research, we will improve the methods and focus more on metabolic processes. An interesting direction will be to assess the aqua fitness training program’s influence on the articular cartilage metabolism and include women with osteoporosis, osteopenia, and history of fractures.

## Figures and Tables

**Table 1:** Somatic features before and after the intervention in the training and control groups.

Parameters		Assessment at Baseline	Assessment at 6 Months
Body mass (kg)	T	71.16 (11.10); 71.50 (65.80–73.70)	71.34 (11.90); 68.30 (64.70–74.30)
	C	77.06 (13.85); 78.70 (66.10–86.10)	77.26 (13.95); 78.70 (65.10–87.00)
BMI (kg/m^2^)	T	27.49 (3.48); 28.28 (24.24–28.60)	27.72 (3.92); 27.00 (25.10–29.00)
	C	28.54 (4.39); 28.08 (24.73–32.55)	28.78 (4.51); 29.00 (25.10–32.50)
Total fat (%)	T	41.07 (5.97); 40.10 (39.30–45.60)	40.46 (6.29); 39.60 (38.50–45.10)
	C	41.28 (6.68); 44.50 (35.80–47.50)	41.29 (6.57); 44.20 (36.00–46.30)
Lean body mass (kg)	T	39.71 (4.42); 40.46 (37.71–41.36)	40.18 (3.89); 40.60 (37.38–41.90)
	C	42.65 (4.14); 41.41 (40.45–45.61)	42.82 (4.35); 41.34 (40.156–44.00)
Total fat (kg)	T	28.30 (7.98); 27.50 (24.39–30.74)	28.10 (8.88); 26.96 (24.49–29.83)
	C	31.22 (10.13); 36.12 (22.32–37.68)	31.31 (10.04); 36.70 (22.06–38.12)
Android fat tissue (%)	T	45.17 (8.14); 45.70 (42.70–51.80)	43.83 (9.41); 44.00 (42.10–50.00)
	C	46.34 (8.16); 44.80 (39.70–54.20)	45.90 (7.72); 45.70 (40.20–53.10)
Gynoid fat tissue (%)	T	48.09 (5.08); 50.10 (44.90–52.00)	46.79 (4.96); 47.70 (43.40–50.50)
	C	45.96 (6.48); 47.30 (42.10–51.30)	45.88 (5.66); 45.90 (43.70–50.20)

Results are expressed as mean (SD); median (interquartile range).

**Table 2:** Areal bone mineral density, femur strength index and bone turnover markers before and after the intervention in the training and control groups.

Parameters		Assessment at Baseline	Assessment at 6 Month
Total BMD (g/cm^2^)	T	1.12 (0.13); 1.09 (1.05–1.21)	1.11 (0.11); 1.09 (1.07–1.19)
	C	1.13 (0.08); 1.14 (1.11–1.16)	1.13 (0.08); 1.14 (1.13–1.14)
BMD L_1_–L_4_ (g/cm^2^)	T	1.05 (0.21); 1.02 (0.91–1.21)	1.04 (0.20); 1.00 (0.87–1.18)
	C	1.11 (0.09); 1.11 (1.08–1.19)	1.11 (0.10); 1.13 (1.05–1.17)
BMD total femur (g/cm^2^)	T	0.95 (0.16); 0.93 (0.83–1.11)	0.94 (0.16); 0.91 (0.83–1.12)
	C	0.94 (0.09); 0.95 (0.90–0.96)	0.94 (0.10); 0.94 (0.89–0.96)
BMD femoral neck (g/cm^2^)	T	0.90 (0.16); 0.88 (0.76–1.08)	0.90 (0.16); 0.88 (0.77–1.06)
	C	0.90 (0.09); 0.92 (0.85–0.98)	0.90 (0.09); 0.93 (0.83–0.97)
Femur strength index (N)	T	1.31 (0.28); 1.30 (1.20–1.50)	1.42 (0.34); 1.40 (1.20–1.70) *
	C	1.27 (0.18); 1.30 (1.20–1.40)	1.19 (0.24); 1.10 (1.10–1.40)
T–score L_1_–L_4_	T	−1.06 (1.73); −1.30 (−2.20–0.20)	−1.19 (1.69); −1.50 (−2.6–0.00)
	C	−0.57 (0.77); −0.60 (−0.90–0.10)	−0.60 (0.81); −0.50 (−1.10–−0.10)
T–score total femur	T	−0.48 (1.28); −0.70 (−1.40–0.80)	−0.52 (1.28); −0.80 (−1.50–0.90)
	C	−0.50 (0.70); −0.50 (−0.90–−0.40)	−0.52 (0.82); −0.50 (−0.90–−0.40)
T–score femoral neck	T	−1.00 (1.18); −1.20 (−2.00–0.30)	−1.01 (1.16); −1.10 (−2.00–0.20)
	C	−1.01 (0.65); −0.90 (−1.40–−0.40)	−1.00 (0.65); −0.70 (−1.50–−0.50)
OC (ng/mL)	T	1.47 (0.67); 1.39 (1.17–1.92)	1.78 (1.00); 1.53 (1.20–2.37)
	C	1.70 (0.70); 1.84 (1.04–2.17)	1.69 (0.80); 1.73 (0.94–2.30)
CTX (ng/mL)	T	0.36 (0.10); 0.30 (0.29–0.37)	0.38 (0.11); 0.35 (0.32–0.42)
	C	0.53 (0.37); 0.44 (0.27–0.66)	0.56 (0.22); 0.61 (0.34–0.73)

Results are expressed as mean (SD); median (interquartile range). * indicates a significant difference (*p* < 0.05).
